# Enhancing EEG Decoding with Selective Augmentation Integration

**DOI:** 10.3390/s26020399

**Published:** 2026-01-08

**Authors:** Jianbin Ye, Yanjie Sun, Man Xiao, Bo Liu, Kele Xu

**Affiliations:** 1 College of Computer Science and Technology, National University of Defense Technology, Changsha 410073, China; jb_ye20@nudt.edu.cn (J.Y.); sunyanjie21@nudt.edu.cn (Y.S.); xiaomannudt@nudt.edu.cn (M.X.); 2 Strategic Assessments and Consultation Institute, Academy of Military Science, Beijing 100091, China; kyle.liu@nudt.edu.cn

**Keywords:** auditory electroencephalography, machine learning, self-supervised learning, automated augmentation

## Abstract

Deep learning holds considerable promise for electroencephalography (EEG) analysis but faces challenges due to scarce and noisy EEG data, and the limited generality of existing data augmentation techniques. To address these issues, we propose an end-to-end EEG augmentation framework with an adaptive mechanism. This approach utilizes contrastive learning to mitigate representational distortions caused by augmentation, thereby strengthening the encoder’s feature learning. A selective augmentation strategy is further incorporated to dynamically determine optimal augmentation combinations based on performance. We also introduce NeuroBrain, a novel neural architecture specifically designed for auditory EEG decoding. It effectively captures both local and global dependencies within EEG signals. Comprehensive evaluations on the SparrKULee and WithMe datasets confirm the superiority of our proposed framework and architecture, demonstrating a 29.42% performance gain over HappyQuokka and a 5.45% accuracy improvement compared to EEGNet. These results validate our method’s efficacy in tackling key challenges in EEG analysis and advancing the state of the art.

## 1. Introduction

Electroencephalography (EEG) signal analysis is a critical area of neuroscience that focuses on studying the brain’s electrical activity as recorded by electrodes placed on the scalp [1,2,3]. EEG plays a dual role in clinical diagnostics and cognitive research, offering valuable insights into neurological functions and disorders. In the clinical domain, EEG is widely recognized for its ability to diagnose conditions such as epilepsy, sleep disorders, and traumatic brain injuries, providing essential information for real-time monitoring and evaluation. Beyond medical applications, EEG has become integral to brain–computer interfaces (BCIs), enabling interaction between users and devices through brain signals, thus driving advancements in assistive technologies and rehabilitation methodologies. As a non-invasive neuroimaging technique, EEG captures cerebral electrical patterns that are indispensable for understanding complex brain activities. The analysis of EEG signals has inspired and continues to benefit from a broad range of methodological advancements across neuroscience [4,5,6,7], signal processing [8,9], and data analysis [10].

**Figure 1:**
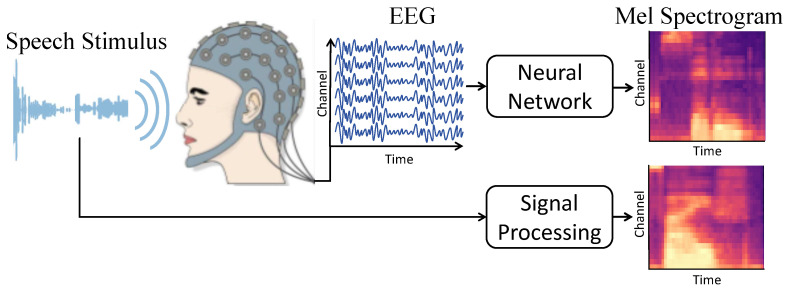
A typical auditory decoding process that extracts meaningful auditory/visual stimulus information from EEG signals, aiding in the exploration of the neural mechanisms underlying speech/vision perception.

EEG auditory decoding is a related research field that aims to decode the features of speech stimuli from EEG. This is achieved by presenting participants with speech while recording an EEG. In this realm, researchers have delved into the intricate, nonlinear relationships between neural activity and observable behaviors, employing machine learning techniques [11,12,13,14,15,16]. These works underscore the versatile applications of EEG in both academic research and practical implementations. Figure 1 demonstrates a conventional EEG auditory decoding procedure, wherein the EEG signals are recorded concurrently with auditory or visual stimuli, followed by the decoding of these signals through diverse decoders.

However, traditional machine learning techniques often encounter difficulties with the high noise levels and temporal variability inherent in EEG signals, necessitating expert oversight for data preprocessing and parameter configuration. Recent advances in deep learning have leveraged its enhanced nonlinear modeling capabilities, facilitating an end-to-end learning approach. For example, Borsdorf et al. [17] make use of a multi-attention mechanism and a gated recurrent neural network to model the temporal dynamics of the EEG signal. Faghihi et al. [18] use a spiking neural network to decode the EEG signals to detect auditory spatial attention. Cai et al. [19] further utilize the spiking graph neural network to capture the dynamic connection between EEG channels. However, this approach’s reliance on fully supervised learning models presents notable constraints. Predominantly, in a conventional fully supervised setting, models are tailored from the ground up for each subject, culminating in a highly individual-specific knowledge base that hampers generalization to new subjects. Furthermore, the efficacy of fully supervised learning heavily depends on the availability of large and high-quality human-annotated datasets. A paucity of such data often leads to diminished model performance, especially within intricate neural network frameworks and novel individuals, thereby compounding the difficulty in crafting versatile and efficient EEG decoding mechanisms.

To address these challenges, we pivot towards self-supervised learning (SSL) as a potent solution. This approach leverages unlabeled data to pre-train deep neural networks, utilizing inherent data features to imbue the model with broader, more generalized knowledge. This foundational training phase is subsequently refined through fine-tuning with a labeled dataset, allowing the network to adjust and improve its performance based on specific, labeled examples. This approach harnesses the vast availability of unlabeled data, enabling more efficient and effective model training by initially learning from the data’s intrinsic patterns before applying targeted adjustments using a smaller set of labeled data. Prior studies have highlighted that self-supervised techniques enhance model robustness and diminish uncertainty [20]. Despite its success in image processing, the adoption of SSL in biosignal processing is less explored, especially in EEG analysis. Initial endeavors in this direction have explored the utilization of diverse augmentation strategies to pre-train encoders to recognize augmented signals, subsequently applying partial or full fine-tuning with labeled data [21,22,23,24,25,26]. While these approaches have yielded promising results, the effectiveness of different augmentation techniques varies across different network architectures and EEG decoding tasks, with some combinations potentially being counterproductive. This observation inspires a novel proposition: could we devise an end-to-end adaptive augmentation method? Such a framework would leverage contrastive learning to bolster the encoder’s representational capacity while autonomously selecting the most effective augmentation combination, thereby maximizing performance.

This paper introduces an end-to-end framework for adaptive augmentation selection, capable of autonomously selecting the optimal augmentation strategies for varying models and tasks. Moreover, we present NeuroBrain, a novel model designed to better effectively decode EEG signals. Our contributions can be summarized as follows:

- We introduce a novel end-to-end framework featuring an adaptive mechanism for the selection of augmentation combinations, specifically engineered for EEG auditory decoding. This innovation facilitates the autonomous determination of the efficacious augmentation strategies across diverse models and tasks.
- To enhance the efficiency of single-model EEG decoding, we introduce NeuroBrain. This model dynamically refines the representation of EEG channels and temporal patterns through an attention mechanism. Combined with a multi-receptive field (MRF) fusion module, it more effectively captures local and global dynamics, leading to improved capabilities in decoding EEG information for both reconstruction and classification tasks.
- Our methodology demonstrates superior performance on two benchmark EEG decoding tasks: SparrKULee for signal reconstruction and WithMe for signal classification. Notably, it surpasses conventional baseline models in both scenarios involving the same subjects (within-subject) and new subjects (unseen-subject), highlighting its effectiveness and generalizability in EEG signal analysis.

The remainder of this paper is organized as follows: Section 2 provides a review of the related literature to contextualize our work within the existing research landscape. Section 3 delineates the proposed methodology in detail. The evaluation of our method, inclusive of a visualization analysis and ablation study, is presented in Section 4. Finally, Section 5 encapsulates a summary and conclusion of this study.

## 2. Background

### 2.1. Auditory EEG Decoding

Auditory EEG decoding techniques aim to decode auditory information from EEG signals, providing a new means of human communication via brain signals. Several studies [9,11,27,28,29,30] have proposed various methods and networks. Pallenberg et al. [31] developed a long short-term memory-based neural network architecture that accurately detects the speaker the person is paying attention to. Lee et al. [32] proposed an automatic speech recognition decoder that contributes to decomposing the phonemes of generated speech, thereby displaying the potential of voice reconstruction from unseen words. Dyck et al. [33] introduced a WaveNet-based model that achieved second place in the Auditory EEG Challenge, demonstrating the effectiveness of their network architecture and training strategies. Fu et al. [34] proposed a novel convolutional recurrent neural network for auditory EEG decoding, outperforming linear and state-of-the-art DNN models. Song et al. [35] introduced a compact Convolutional Transformer called EEG Conformer, which achieved state-of-the-art performance and has great potential as a new baseline for general EEG decoding. Thornton et al. [36] focused on the match–mismatch subtask, classifying which speech segment aligned with the EEG signals, and proposed a solution using short temporal EEG recordings. Sun et al. [30] introduced the concept of delay learning [37], which explicitly incorporates the temporal delay between a stimulus and its corresponding EEG pattern. This enhancement significantly improved performance by aligning the EEG signals more precisely with the associated stimuli.

Our research aims to enhance auditory EEG decoding by innovatively applying self-supervised learning techniques, while the primary network architecture is based on the encoder–decoder model.

### 2.2. Self-Supervised Learning for EEG

Self-supervised learning has been widely used in many fields [38,39,40,41]. In natural language processing, self-supervised models like BERT (Bidirectional Encoder Representation from Transformers) [42] can be well applied to language modeling tasks. In computer vision, self-supervised models like MAE (Masked Autoencoders) [43] can be applied to image representation learning. In the EEG signal processing field, some endeavors have been made. He et al. [44] proposed a self-supervised learning-based channel attention MLP-Mixer network for motor imagery (MI) decoding with EEG, which effectively learned long-range temporal information and global spatial features. Ma et al. [45] introduced a self-supervised learning CNN with an attention mechanism for MI-EEG decoding, demonstrating that learning temporal dependencies improved performance. Wang et al. [46] developed an end-to-end EEG decoding algorithm using a low-rank weight matrix and principled sparse Bayesian learning, achieving improved performance under a self-supervised framework. Partovi et al. [47] proposed a self-supervised deep learning architecture that learned a common vector representation of EEG signals, successfully distinguishing binary classes in different BCI tasks. Song et al. [48] demonstrated the feasibility of learning image representations from EEG signals using a self-supervised framework, with attention modules capturing spatial correlations. Acknowledging the validity of self-supervised learning, we innovatively apply it to address challenges in auditory EEG decoding.

### 2.3. EEG Signal Augmentations

Various data augmentation techniques have been proposed to generate additional training samples for EEG-based applications. Bhaskarachary et al. [49] explored using machine learning models for EEG preprocessing, feature extraction, and classification to diagnose autism spectrum disorder (ASD). Liu et al. [50] proposed a spatial–temporal data augmentation scheme for brain disorder classification and analysis. Jiang et al. [22] designed various augmentations, including noise addition and affine transformation, which achieve competitive performance on sleep staging tasks. In the EEG-based emotion recognition task, various data augmentation methods have been proposed [21,23,25], which achieved satisfactory emotion recognition performance. We adopted the above method in our work.

However, the above augmentations share a familiar obstacle: implementing comprehensive augmentation policies requires costly manual design and combined efforts. To address this obstacle, Cu-buk [51] presented the AutoAugment algorithm in a computer, which uses reinforcement learning to discover transformations and parameters for data augmentation. This algorithm automatically looks for a suitable data augmentation policy. However, some improvements have been proposed due to the high computational costs and limitations of offline searching in AutoAugment. For instance, Lim [52] uses Bayesian optimization instead of reinforcement learning to reduce the computational burden. Inspired by Lim’s work, we utilize Bayesian optimization to speed up the search process. PBA [53] introduces the concept of population thinking and uses genetic algorithms to find the best strategy from a set of data augmentation strategies. This method achieves dynamic updating of augmentation strategies and continuously optimizes model performance. Lin [54] proposes trainable data augmentation strategy parameters and uses gradient-based optimization to overcome the limitations of offline searching. This method dynamically updates the strategy during training based on model feedback and performs well. Hataya et al. [55] propose Faster AutoAugment, which uses automatic differentiation techniques to search for data augmentation strategies during training. This method achieves efficient augmentation by learning the algorithm to search for augmentation strategies, allowing the learned strategies to be shared and updated throughout the training process. Sun et al. [56] proposed Audio Automatic Augmentation, which extends the AutoAugment method to the audio signal field. Ref. [57] proposes a proxy learning curve for the Bayes classifier for estimation of the minimum training sample size for a required performance. However, there is a lack of research on designing suitable augmentations for the EEG signal. Our research represents the first attempt to develop automatic methods for enhancing EEG signals, significantly improving auditory EEG decoding performance.

## 3. Methodology

### 3.1. Overview of Framework

**Figure 2:**
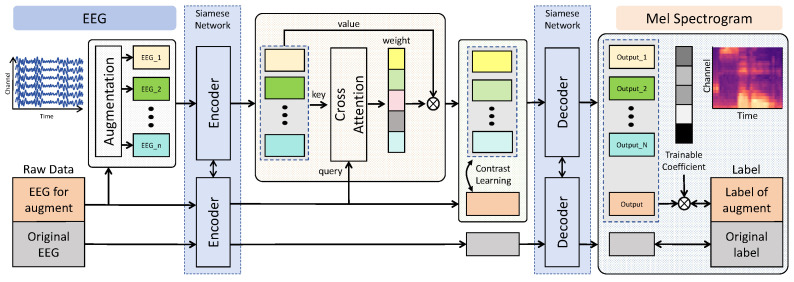
End-to-end enhancing EEG Siamese network framework with selective augmentation integration. In the beginning, raw EEG data is split into two parts for enhancing training and simple training with the Siamese network. Then, we introduce a cross-attention module and contrast learning to align enhanced embedding and original embedding by learning their similarity. Finally, we adaptively select a better integration via a trainable coefficient and then calculate Pearson correlation values of pairs of auditory EEG data.

The schematic of our proposed EEG augmentation framework is illustrated in Figure 2, encompassing three stages: (1) augmentation for EEG signal to form enhanced embedding, (2) self-supervised learning with enhanced embedding, and (3) augmentation integration selection. Firstly, to address the issue of insufficient and inherent interference of EEG data, we conducted data augmentation on a subset of the EEG dataset. The raw EEG data is segregated into two streams. The original part is directly routed to the encoder and decoder without additional processing, while the other part undergoes data augmentation, which is illustrated in Section 3.1.1. The EEG signal is enhanced by each augmentation technique, and then, the enhanced EEG signals are delivered to the encoder. A Siamese network [58] is introduced by delivering enhanced EEG data and original EEG data to the encoder/decoder. The encoder in the Siamese network assimilates the representation of both the enhanced and original data. Secondly, given two kinds of feature representation, the embedding after augmentation followed by the encoder should be aligned to the embedding from raw data. After obtaining the weights of the feature map, we protrude the area they look like and pay less attention to the different parts that may be produced by noise signals. Expecting that enhanced embedding is similar to raw embedding as much as possible, we adopt contrast learning here to pull them closer, which is mentioned in Section 3.1.2. Although SSL tries to align various enhanced features, it is still a challenge to bond stylized augmentation techniques. Aiming at the mutual exclusion problem of augmentation techniques, in Section 3.1.3, we designed a selective augmentation integration strategy to adaptively search for the best integration policy of data augmentation methods during training. With negative Pearson correlation loss backward propagating, the importance of a certain integration is reflected by a set of trainable coefficients. According to the importance coefficient of these enhanced outputs, we remove worse outputs and retrain again with new augmentation integration.

#### 3.1.1. Data Augmentation Techniques

**Figure 3:**
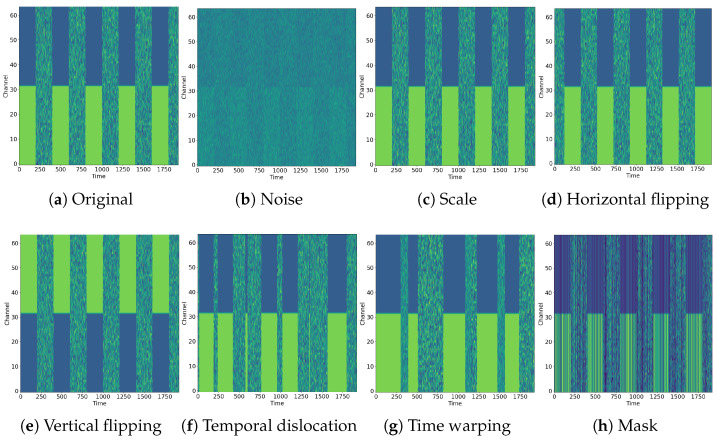
Visual exemplars pertaining to the augmentation techniques. (**a**) An example of original test EEG data, (**b**–**h**) The EEG data after the corresponding augmentation techniques are applied to the original EEG data.

Since the collected EEG signals are easily disturbed by ambient noise and the subject’s irregular brain activity is unrelated to the experiment, and the acquisition cost is high, it is necessary to perform data augmentation to make the model learn the characteristics of EEG signals such as time, noise, and other dimensions, improving the robustness of the model. In our training framework, we incorporate seven signal transformation methods that are commonly used in prior research [21,23,25], which are visualized in Figure 3 and described below.

- Noise: A random noise segment is collected from the Gaussian distribution and added to the EEG signal.
- Scale: We perform a range of amplifications or reductions in the amplitude of the EEG signal. The scaled range is constrained from 0.5 to 2.
- Horizontal flipping: We perform a horizontal flip of two-dimensional EEG data along an intermediate time point, which is equivalent to putting the EEG data in reverse order.
- Vertical flipping: We perform a vertical flip of two-dimensional EEG data along an intermediate channel, which is equivalent to reversing the EEG data on the channel dimension.
- Temporal dislocation: We divide the EEG signal into 10 equal-length segments in order of timeline and then randomly shuffle these segments and reassemble them.
- Time warping: We divide the EEG signal into 10 segments of equal length according to the order of the timeline and then randomly select half of the segments to stretch in the time dimension and the other half to telescope in the time dimension. In this paper, the strength of the stretch is between 1.5 and 2 while the telescoping ranges from 0.5 to 0.67.
- Mask: A random subset of EEG signal is masked out and replaced by mask tokens initialized by zeros. In this paper, the masking ratio is 30%, leaving 36,864 out of 122,880 (1920 × 64) data points.

#### 3.1.2. Self-Supervised Learning

Data augmentation endows EEG signals with a variety of manifestations, improving the robustness of the encoder. But, for the decoder, multiple kinds of feature maps from the same EEG signal should lead to the same output. Misalignment of the feature map may lead to an inexact reconstructed result. Thus, we utilize contrast learning to make the embedding from the enhanced EEG signal similar to the embedding from the original one. Followed by CLIP [59], a contrast learning loss is computed for each pair of embedding. We assume that there are *n* participating augmentation methods and every augmentation method corresponds to one embedding $f_{i},i\in \left\{1,2,3,\ldots ,n\right\}$, which composites with the original embedding $f_{a}$ as a pair. Then, a scaled pairwise cosine similarity is computed as follows:

(1) $$logits_{i}=f_{a}\cdot f_{i}^{T}\cdot \tau$$

where τ is a trainable temperature parameter controlling the range of logits. Given the similarity of the two embeddings, the contrast loss can be obtained by

(2) $$L_{cont_{i}}=L\left(logits_{i},K\right)$$

Let *L* be the cross entropy loss function and *K* be a list of [1,2,…,k], where *k* is equal to the length of logits. After that, we obtain an average contrast loss $L_{cont}$ from *n* pairs of embedding by

(3) $$L_{cont}=\frac{\Sigma_{i=1}^{n}\left(L_{cont_{i}}\right)}{n}$$

#### 3.1.3. Selective Augmentation Integration

Data augmentation techniques for EEG signal described in Section 3.1.1 are beneficial to the robustness and generalization of the EEG decoder. However, applying all augmentation techniques is not fit for all models leading to worse performance. To this end, we propose selective augmentation integration, which is an adaptive training paradigm by updating the augmentation integration policy.

Algorithm 1 illustrates the selective augmentation integration process, which includes two main stages. The first stage is responsible for fouling out the useless one. We maintain an augmentation method set A that contributes a lot to the model *M* used. We train and evaluate *M* from scratch with A in every loop and update the weight coefficient vectors *w* for each technique in *A*. During training, *w* is constantly monitored, and once one of them falls below the threshold, its corresponding method is removed from the current integration policy *A*. After finishing a total training period, the result for the current augmentation integration policy is saved. After that, we figure out the distribution of *w* to erase the augmentation technique with a low outlier from A. When all weight coefficient values in *w* are evenly distributed, we fix the best augmentation integration policy for the next stage.

Each augmentation technique has a unique enhancing operation. Nevertheless, different enhancing operations are not all orthogonal with inevitable distractions to each other. Due to the existence of potential obstacles between augmentation techniques, applying a single augmentation technique may achieve a better performance. The second stage is responsible for evaluating performance for every single augmentation method in A to try to eliminate the disturbance between different techniques. For each latent efficient augmentation method in A, we train the model individually and save the result as an alternative. Finally, given the results from integrated and single augmentation techniques, we select the model with the best performance naturally.
**Algorithm 1** The flowchart for selective augmentation integration.Input:Augmentation method set A, Training data $D_{train}$, Evaluate data $D_{val}$, Model *M*, weight coefficient *w*, threshold *t*Output:Trained model $\hat{M}$1:R⟵{};2:**while** True **do**3:   *A*⟵A;4:   initialize *w*;5:   **for** epoch=1 to Max epoch **do**6:     $\hat{M}$⟵ Train *M* using $D_{train}$ with *A*;7:     Evaluate $\hat{M}$ using $D_{val}$ and update *w*;8:     **for** i=1 to number of *A* **do**9:        **if** $w_{i}<t$ **then**10:          $A=A-A_{i}$;11:      **end if**12:    **end for**13:   **end for**14:   **if** There is $w_{i}$ significantly lower than other values in *w* **then**15:      $A=A-A_{i}$;16:   **else**17:      break;18:   **end if**19:**end while**20:**for** *i* to number of A **do**21:   **for** epoch=1 to Max epoch **do**22:     $\hat{M}$⟵ Train *M* using $D_{train}$ with $A_{i}$;23:     Evaluate $\hat{M}$ using $D_{val}$;24:   **end for**25:   R = $R⋃\hat{M}$;26:**end for**27:**return**max(R)

#### 3.1.4. Loss Function

For the auditory EEG regression task, in this paper, we use negative Pearson correlation loss and L1 loss to measure the similarity between the reconstructed mel-spectrogram and the original mel-spectrogram. We calculate their Pearson correlation as follows:

(4) $$L_{P}=\frac{E[\left(X-\mu_{X}\right)\left(Y-\mu_{Y}\right)]}{\sqrt{\Sigma_{i=1}^{n}{\left(X_{i}-\mu_{X}\right)}^{2}}\sqrt{\Sigma_{i=1}^{n}{\left(Y_{i}-\mu_{Y}\right)}^{2}}}$$

Thus, the final training loss is given as follows:

(5) $$L=L_{P}+\alpha *L_{cont}+\beta *L_{1}$$

where α and β are optimal value. In this paper, we experimentally set the optimal value of α and β to 0.01 and 0.001, respectively.

For the classification task, Cross-Entropy Loss is employed to quantify the discrepancy between the actual labels and the predicted outputs. Consequently, this loss is formulated as follows:

(6) $$L_{C}=-\frac{1}{N}\sum_{i=1}^{N}\sum_{c=1}^{C}y_{i,c}\log\left({\hat{y}}_{i,c}\right)$$

Herein, *C* symbolizes the total count of categories, while *N* denotes the total number of samples. In this context, *y* represents the actual labels, and $\hat{y}$ signifies the predicted probabilities. In alignment with our proposed methodology, the final training loss is formulated a followss:

(7) $$L=L_{C}+\alpha *L_{cont}$$

### 3.2. Proposed Reconstruction Model for Auditory EEG

**Figure 4:**
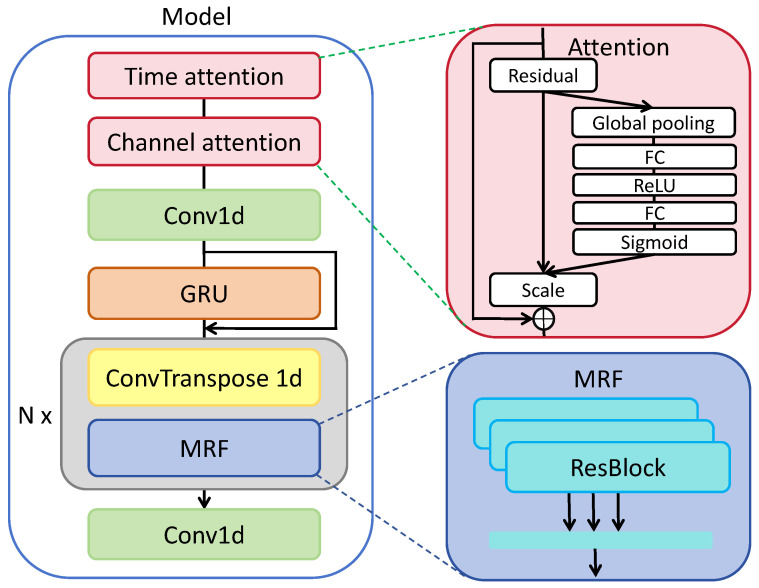
Illustration of NeuroBrain model.

For reconstruction and classification tasks, we designed a NeuroBrain model adapted to the proposed framework as the encoder and the decoder. We implemented our model based on NeuroTalk [32]. Figure 4 illustrates the details of our model. The encoder consists of all modules except the last convolution layer, which we treated as the decoder corresponding to our proposed framework. Firstly, we introduced attention mechanisms on the time and channel dimensions to dig out the importance of different time points and channels. Specifically, SENet [60,61] was adopted as the attention module. It consists of squeeze, excitation, and scale, three key operations. Global average pooling is generally used to squeeze the feature map. After that, in the excitation part, two full connection layers were used to figure out the weights of different timelines or channels. Then, we multiplied the activation value of the weights by the feature map. Secondly, the feature map went through a 1D convolution layer (Conv1d), extracting spatial information, and a gated recurrent unit (GRU), extracting temporal information. Thirdly, *N* transposed convolutions and a multi-receptive field fusion (MRF) module that consists of three residual blocks with multiple kernel sizes was applied to extract the feature map. After that, we obtained an embedding vector and put it into a 1D convolution as a decoder.

## 4. Experimental Results

In this section, we evaluate the efficacy of the proposed adaptive augmentation selection and contrastive learning paradigm across two EEG decoding tasks: reconstruction and classification. We selected several widely used models for benchmarking and compared their performance with and without the integration of our algorithm.

### 4.1. Implementation Details

For both datasets, we set the learning rate to 0.0005 and the max training epoch to 100, unless explicitly stated. All experiments were optimized using the AdamW [62] algorithm. Meanwhile, we used an early-stop mechanism with a max patience of 6 to reduce cost. We experimentally left 10% training data for augmentation. We conducted our experiments on four GeForce GTX 3090 Ti GPUs.

### 4.2. Dataset

#### 4.2.1. SparrKULee

We trained and evaluated our methods on the official dataset SparrKULee [63], sourced from the Auditory EEG Challenge—ICASSP 2024. The SparrKULee dataset comprises 168 h of auditory EEG data with speech stimuli, with each stimulus lasting between 90 and 150 min. A cohort of 85 individuals, native Dutch speakers, participated in the EEG experiment within a controlled laboratory setting. In this paper, the EEG data was downsampled to 64 Hz and was divided into shorter 30 s intervals aligned with the corresponding speech stimuli, resulting in a total of 14,889 samples. We split the dataset into training, validation, and testing subsets in a ratio of 7:2:1.

Acquisition setup. Electroencephalographic (EEG) data were acquired using a BioSemi ActiveTwo system equipped with 64 active Ag-AgCl electrodes. The recording setup included integrated Common Mode Sense (CMS) and Driven Right Leg (DRL) reference electrodes, supplemented by bilateral mastoid electrodes. All electrodes were mounted on a BioSemi head cap arranged according to the international 10–20 system. Prior to electrode placement, each participant’s cranial dimensions were measured from nasion to inion to ensure proper cap sizing. Mastoid regions were prepared with Nuprep abrasive gel followed by alcohol cleansing to optimize skin–electrode contact. The electrode cap was adjusted to maintain equal distances between the nasion and Cz electrode, inion and Cz, and left/right preauricular points relative to Cz. Continuous EEG signals were digitized at 8192 Hz using BioSemi ActiView software. During recording, participants maintained a seated position with eyes open. Electrode offsets were monitored throughout the session and maintained within ±20 μV through periodic application of additional conductive gel when necessary. All data were stored for offline analysis.

In this paper, for EEG and speech stimuli, we applied preprocessing procedures provided by Accou et al. [63], which are commonly used to mitigate noise and eliminate artifacts.

#### 4.2.2. WithMe

The WithMe dataset, derived from the Withme experiment [64], encompasses EEG recordings from 42 participants. This experiment facilitates an investigation into the cognitive mechanisms of attention and memory within the context of human–computer interaction, through the observation of varied sequences of stimuli. Participants engage with video sequences and audio cues, each extending for either 10 or 16 s in duration, designed to elicit cognitive engagement through auditory cues or targeted numerical stimuli, thereby challenging the participants to retain these numerical sequences in memory. The dataset comprises a total of 120 distinct sequence groups, providing a robust framework for analyzing the intricate interplay between cognitive stimulation and memory retention in the realm of human–computer interfaces. While the original experiment focused on the effects of attention and rhythmic audio/visual stimuli as measured by a behavioral questionnaire, it could be valuable to directly explore and analyze the corresponding EEG data to gain deeper insights into these cognitive processes. The corresponding processed data are also introduced here [65].

For our study, we selected 38 participants for training and internal testing, dividing their data into training and testing sets—known as within-subjects. The data from the remaining four participants served to assess our model’s generalizability, termed unseen subjects. Specifically, we partition the Withme data into a training set and two testing sets: one for within-subject evaluations, consisting of 18,176 training instances and 4580 validation instances, and another for unseen-subject assessments, with 2400 validation instances. Preprocessing the EEG data involved re-referencing each channel to the average activity of the mastoid electrodes, band-pass filtering between 1 and 30 Hz, and downsampling to 64 Hz. We then segmented the data into 1.2 s epochs based on trigger events, with the final preprocessing step normalizing the EEG channel data to ensure zero mean and unit variance for each sample.

Acquisition setup. All experimental sessions were conducted in an electromagnetically shielded and sound-attenuated booth to ensure optimal signal quality. Participants were seated in an adjustable chair positioned at a fixed viewing distance of 1.5 m from a 20-inch Nikkei NLD20MBK display (NIKKEI, Tokyo, Japan). The monitor, with a refresh rate of 50 Hz, was aligned at eye level to maintain consistent visual angle across participants and to ensure precise synchronization of the 200 ms audiovisual stimuli. Auditory stimuli were routed through an RME Fireface UCX soundcard configured for dual-channel output. One channel delivered the sound stimulus, while the second transmitted a synchronization trigger signal. The stimulus channel was duplicated for binaural presentation via ER-2 insert earphones (Etymotic Research), with all sounds calibrated to 70 dBSPL.

### 4.3. Evaluation Methods and Metrics

To make a fair comparison, we implemented various well-acknowledged reconstructions and classification methodologies pertinent to EEG analysis. In the context of the reconstruction endeavor, our approach encompassed the following. (1) Linear: it consists of one convolution layer and one full connection layer. (2) VLAAI [66]: it is a subject-independent speech decoder using ablation techniques, which is mainly composed of convolution layers and fully connected layers. (3) HappyQuokka [67]: it is a pre-layer normalized feedforward transformer (FFT) architecture utilizing the Transformer’s self-attention mechanism and designing an auxiliary global conditioner to provide the subject identity. (4) FastSpeech2 [68]: Transformer-based FastSpeech [69] architecture has been proven to be efficient in HappyQuokka for auditory EEG regression tasks. FastSpeech2 handles the one-to-many mapping problem in non-autoregressive TTS better than FastSpeech by introducing some variation information of speech. We only used FastSpeech2’s decoder as an evaluation model in this paper. (5) NeuroBrain: as shown in Figure 4, for the reconstruction task, we used three transposed convolutions. For the classification tasks, we employed the following methods: (1) EEGNet [70], a compact convolutional neural network known for its effectiveness in EEG decoding, and (2) Neurobrain, where our implementation is simplified to utilize just a single transposed convolution layer.

To evaluate reconstruction results and classification results comprehensively, the following metrics were introduced to measure effectiveness.

Pearson correlation: Pearson correlation is a statistical measure that quantifies the strength and direction of the linear relationship between two variables X and Y, which is commonly used in baselines [66,67,68]. Pearson correlation coefficient pc can be expressed as

(8) $$pc=\frac{n(\sum XY)-(\sum X)(\sum Y)}{\sqrt{\left[n\sum X^{2}-{\left(\sum X\right)}^{2}\right]\left[n\sum Y^{2}-{\left(\sum Y\right)}^{2}\right]}}$$

where *n* denotes the number of data points. A high pc indicates a positive linear relationship between reconstructed speech and real speech stimuli.

Accuracy: This metric quantifies the percentage of correctly predicted samples out of the total number of instances evaluated, thereby providing a straightforward and intuitive measure of the model’s predictive capability. We utilize this metric to assess and compare the efficacy of various models in classification tasks. It can be mathematically represented as follows:

(9) $$Accuracy=\frac{TP+TN}{N}$$

Here, TP denotes True Positives, which are the positive instances correctly identified, and TN denotes True Negatives, referring to the negative instances that have been correctly identified. *N* represents the total number of samples evaluated.

SSIM: The Structural Similarity Index (SSIM) is a widely used quality assessment metric that measures the similarity between two two-dimensional matrices *x* and *y*. It takes into account not only the similarity in terms of point values but also their structures and luminance. It is formulated as follows:

(10) $$SSIM\left(x,y\right)=\frac{\left(2\mu_{x}\mu_{y}+c_{1}\right)\left(2\sigma_{xy}+c_{2}\right)}{\left(\mu_{x}^{2}+\mu_{y}^{2}+c_{1}\right)\left(\sigma_{x}^{2}+\sigma_{y}^{2}+c_{2}\right)}$$

where $\sigma_{x}^{2}$ and $\sigma_{y}^{2}$ are the variances of *x* and *y*, $\sigma_{xy}$ is the covariance, $c_{1}$ and $c_{2}$ are variables to stabilize the division with weak denominator.

CW-SSIM: The Complex Wavelet Structural Similarity Index (CW-SSIM) [71] is an extension of the SSIM index, designed to provide a more comprehensive assessment of image similarity by incorporating the complex wavelet transform. CW-SSIM is less sensitive to unstructured geometric distortion transformations such as scaling and rotation than SSIM and more intuitively reflects the structural similarity of two matrices. CW-SSIM is given by

(11) $$CW-SSIM\left(c_{x},c_{y}\right)=\frac{2\left|\sum_{i=1}^{N}c_{x,i}c_{y,i}^{*}\right|+K}{\sum_{i=1}^{N}{\left|c_{x,i}\right|}^{2}+\sum_{i=1}^{N}{\left|c_{y,i}\right|}^{2}+K}$$

where $c_{x}$ and $c_{y}$ denote two sets of coefficients extracted at the same spatial location in the same wavelet subbands of the two matrices being compared, $c^{*}$ is the complex conjugate of *c*, and *K* is a small positive constant to improve the robustness of the CW-SSIM measure.

It is noteworthy that due to the high level of noise inherent in EEG data and the challenges associated with reconstructing it into audio format, existing studies have reported relatively low Pearson correlation coefficients (below 0.1) in the task of audio reconstruction from the SparrKULee dataset. For a more intuitive comparison, the Pearson correlation coefficient values, SSIM, and CW-SSIM metrics in the table are multiplied by 100 in this paper.

### 4.4. Comparison Experiments for Auditory EEG

In real-world scenarios, the deployed auditory EEG decoders are applied to decode EEG signals from unseen subjects. Therefore, we evaluate all models on the test subset, where the subjects never appear in the training subset. We first evaluate the performance of reconstructing speech signals from raw EEG signals and enhanced EEG signals on the SparrKULee dataset. Table 1 illustrates that our approach significantly outperforms other baseline models. Specifically, our proposed model achieves a Pearson correlation of 0.07135 when incorporating noise augmentation, representing a remarkable improvement of over 29% compared to the best baseline model.

Furthermore, we explore the impact of augmentation methods. Although scale shows efficiency for most models except HappyQuokka, not all augmentation methods contribute to the models. For instance, while noise proves beneficial to our approach, it is harmful to other baseline models, and horizontal flipping diminishes the performance of all models to varying degrees. Therefore, it becomes evident that a simple ensembling of seven augmentation methods could potentially lead to a reduction in performance. Each model may have a preference for specific augmentation methods while exhibiting an aversion to others. For instance, our approach benefits most from noise, while VLAAI prefers scale and mask. Notably, HappyQuokka excels in integrating all augmentation methods. Hence, we need to ascertain the optimal augmentation integration strategy for each model.

For the WithMe dataset, which focuses on distinguishing between target stimuli and distractors in EEG signals, our findings are consistent. As depicted in Table 2, based on Monte Carlo stability experiments, our model notably outperforms the conventional EEGNet, achieving improvements of 5.45% for seen subjects and 1.79% for unseen scenarios. Moreover, the proposed method outperforms others not only in overall classification performance but also in terms of the range between the maximum and minimum values (narrower interval), demonstrating that the selective approach exhibits stronger stability.

We employed the SPSS (version: IBM SPSS Statistics 29.0) data analysis platform to conduct statistical tests to evaluate the effectiveness based on the WithMe dataset. The statistical tests were performed on the values of TPR, TNR, FPR, and FNR, corresponding to Table 3. Such results illustrate the relationship between observed (true) values and expected (predicted) values. The analysis encompassed three complementary statistical approaches: the chi-square test, correlation coefficient analysis, and quantitative strategy testing. The chi-square test is primarily used for categorical variables to assess whether two variables are associated or independent. A larger chi-square value indicates a stronger association between the two variables. Correlation coefficient analysis quantifies the linear relationship between the true labels and the predicted labels. A coefficient close to 1 suggests that the predicted values closely approximate the true values, whereas a coefficient near 0 indicates weak correspondence. The quantitative strategy test further serves as a statistical method to measure the quantitative relationship between predictions and actual outcomes. Together, these tests provide a comprehensive assessment of the model’s performance and its alignment with empirical observations.

In the chi-square test, we computed the Pearson chi-square statistic, likelihood ratio, linear association, and asymptotic significance (*p*). For the correlation analysis (symmetrical measurement), corresponding metrics were derived, including Cramer’s V, the contingency coefficient, Gamma, Spearman’s rank correlation, Pearson’s correlation coefficient, and Cohen’s kappa. Furthermore, as part of the quantitative strategy, we conducted auxiliary analyses involving Lambda, the Cochran–Mantel–Haenszel statistic, the uncertainty coefficient, and Somers’ d value. From statistical test results, we can observe that all data augmentation methods exhibit highly significant statistical associations on the WithMe dataset (*p* < 0.001). Among them, the selective augmentation method demonstrates the best performance across all metrics, achieving the highest values in Pearson’s chi-square (962.5), likelihood ratio (812.7), and linear association (295.1), along with optimal correlation coefficients such as Cramer’s V (0.905), Spearman’s correlation (0.985), and Gamma coefficient (0.999). This highlights the superiority of the selective method in enhancing model discriminative capability. The mask augmentation and vertical flipping methods rank second and third, respectively, while time warping, though still statistically significant, shows relatively lower values across all indicators, suggesting its limited effectiveness in augmentation. Overall, there is a clear gradient in the effectiveness of different augmentation strategies, with selective augmentation, mask augmentation, and spatial transformation methods (vertical/horizontal flipping) performing better, whereas noise addition and temporal transformation methods exhibit relatively weaker enhancement effects. These findings provide empirical evidence for selecting appropriate data augmentation strategies tailored to specific tasks.

Adaptive selection for augmentation techniques. We show the weight coefficient of seven augmentation methods for the SparrKULee dataset during training in Table 4. We observe that the weight coefficients of horizontal flipping and temporal dislocation are significantly lower than other augmentation methods in most models, which means the performance gain from these two augmentation techniques is negligible or even harmful. It is worth noting that horizontal flipping and temporal dislocation both disturb the temporal information of EEG signals, resulting in difficulty in reconstructing the right speech stimuli from out-of-order EEG data. Moreover, time warping only adjusts the length of the timeline but does not destroy the order of segments. Therefore, disturbance to the time dependency is not conducive to reconstructing speech from EEG signals.

Due to the little contribution to regression models, horizontal flipping and temporal dislocation should enjoy a low priority and even be kicked out manually during deployment. Similarly, vertical flipping seems useless to FastSpeech2 on the SparrKULee dataset. To explore an appropriate threshold, we evaluate varying threshold values that adaptively ignore enhanced output with low coefficient values. We do not take horizontal flipping and temporal dislocation into account in this experiment. Table 5 indicates that most models fit well when the threshold is set to 0.4, where four models outperform the original ones while only two models achieve better performance under other threshold values. This suggests that removing inapplicable augmentation techniques is useful for our framework.

We make a fair comparison for augmentation integration selection strategies on SparrKULee. The results in Table 6 demonstrate significant advantages of our adaptive selection policy. Because neither do all augmentation techniques bring improvements for all EEG reconstruction models nor is aggregating any combination of augmentation techniques efficient, it may be unsatisfactory to use a single augmentation method or to merge all enhancing ways. Especially, integrating all augmentation techniques is a disaster for VLAAI and FastSpeech2. It seems reasonable to manually select a group of integration that looked like the best. But, it is still not the best strategy most of the time. There is non-negligible defectiveness under the “Manual” strategy for most models except our approach. This suggests that there may be mutual hindrances between individual augmentation methods that perform well, which is a cost to figure out manually. By contrast, our adaptive selection strategy works for all models since it keeps taking one step forward to search for a better integration. According to the result of our strategy, the best performance of linear and HappyQuokka comes from some integration of augmentation techniques, which for VLA-AI, FastSpeech2, and our approach comes from one single augmentation method. The diversity of results sources illustrates the necessity of adaptive selection.

**Figure 5:**
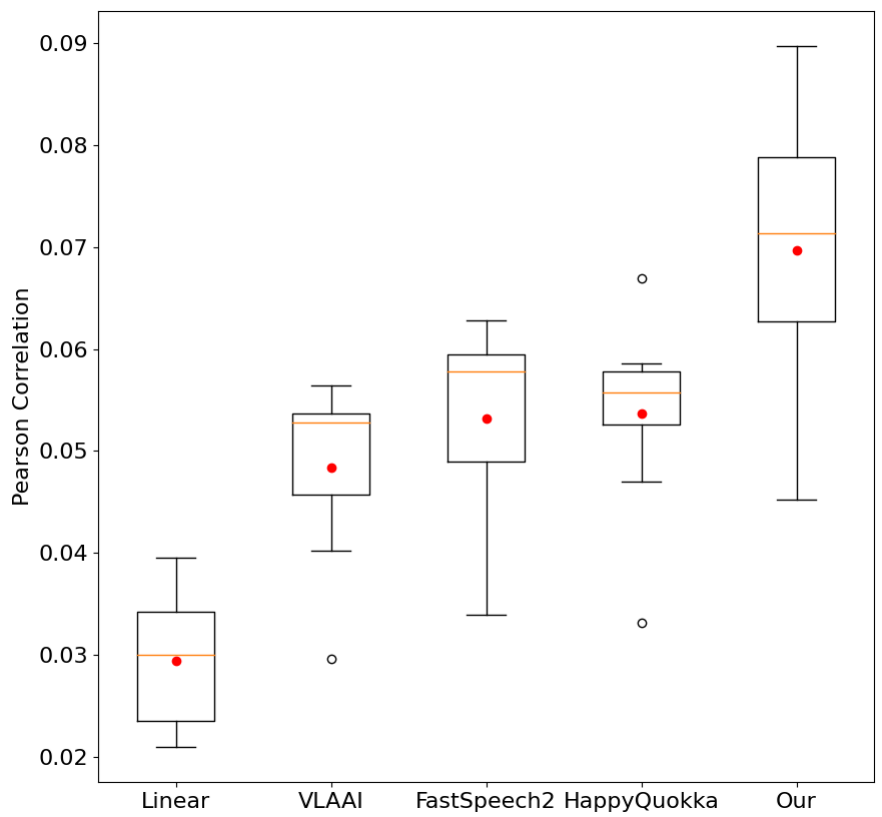
Details of Pearson correlation scores for different subjects on SparrKULee. The colored lines and red points in the boxes represent the models’ median scores and average scores, respectively.

We compared our approach and baseline Pearson correlation scores across eight different subjects in the test subset. The results in Figure 5 visually display excellent generalization and greater robustness of our proposed method over baselines. Our approach contains a high Pearson correlation when meeting EEG data from unseen subjects. This test performance suggests that our model reconstructs speech stimuli from EEG signals with higher accuracy and better diagnoses the subjects’ brain activity.

In the case of the Withme task, our observations indicate that except for “noise” augmentation, which slightly detracts from the performance of the EEGNet model, almost all augmentation techniques individually enhance performance relative to the baseline (original). Intriguingly, when employing the selective augmentation algorithm, the combination of “noise”, “scale”, and “time warping” augmentations yields the most substantial improvement for the EEGNet model. This suggests that although individual augmentations might negatively impact decoding performance, their combination with other augmentations can mitigate these adverse effects and leverage their benefits. Furthermore, for our model, NeuroBrain, when applying selective methods, we find that the ’Mask’ augmentation alone delivers the best performance.

We selected 30%, 70%, and 100% samples from the SparrKULee dataset for the training set and evaluated their regression performance on the validation set. As shown in Table 7, as the number of training samples increases, the model’s decoding performance improves significantly. Because auditory EEG decoding is a challenging task, learning adequate EEG-to-speech features from only a small amount of data is difficult. Even when 100% of the training data are used, performance reaches only 0.1235, underscoring the critical role of data augmentation in auditory EEG decoding. It is noted that the standard deviation is up to 0.0368 when the sample size is equal to 70%. With 70% of the training samples, one split already achieves a score above 0.11, essentially on par with the performance obtained from the full 100% dataset. Thus, training the EEG decoding model on roughly 70% of the data is expected to yield near-optimal results.

**Figure 6:**
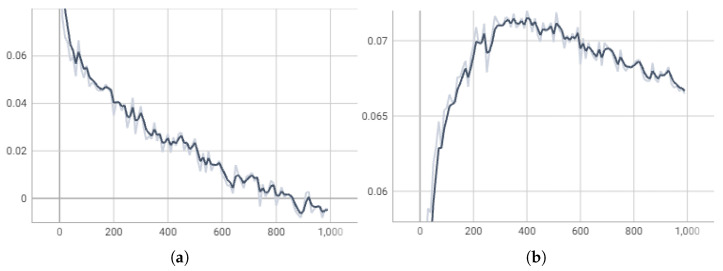
The loss for the train set and Pearson coefficient for the validation set through training steps. (**a**) Training loss; (**b**) validation Pearson coefficient.

We plot the training loss and Pearson correlation for the validation set through training steps, as shown in Figure 6. During the first 400 training steps, the training loss decreases steadily while the Pearson correlation on the validation set improves. After 400 steps, however, although the training loss continues to drop, the Pearson correlation on the validation set begins to decline. This indicates that our model started to overfit the training data beyond 400 steps, leading to degraded performance on the validation set. Therefore, we kept the checkpoint at step 400 as the final model.

### 4.5. Ablation Study

We first make a comparison between our approach and the baseline. Here, we use HappyQuokka as the baseline model and calculate the performance improvement of each variant over the baseline. We then evaluate each variant on SparrKULee. The results shown in Table 8 state that our approach obtains a 29.42% improvement in Pearson correlation compared to the baseline. The results of all cases are worse than our whole framework. The performance without cross-attention and adaptive selection is the worst, which suggests that consistency of enhanced features and adaptive integration has a great impact on the framework. The performance drops to 0.307 without the cross attention mechanism, which means that the attention modules capture temporal and channel dependencies from EEG data effectively. The degrading Pearson correlation without augmentation and contrast learning denotes that augmentation techniques boost the model’s generalization, and contrast learning is useful for feature alignment. In general, augmentation methods and optimization are important for EEG signal processing and applications.

### 4.6. Visualization

**Figure 7:**
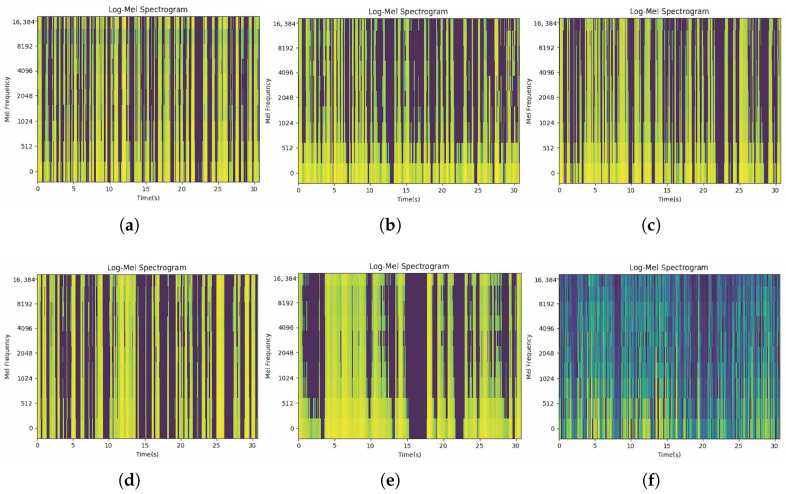
Reconstructed mel-spectrogram from the same EEG signal and the ground-truth speech stimuli. (**a**) Linear; (**b**) VLAAI; (**c**) FastSpeech2; (**d**) HappyQuokka; (**e**) NeuroBrain; (**f**) Ground-truth.

To visually depict the performance, we present reconstructed speech stimuli for five models. To make a fair comparison, these five models take the same EEG signal as inputs. As shown in Figure 7, our approach has a significant advantage in capturing temporal and spatial information from EEG data. We observe a gap in the high-frequency region before 5 s and two prominent silent segments around 20 s. Compared with other methods, our proposed method reproduces the gap before 5 s and the adjacent silent region near 20 s. It demonstrates that our approach better captures the temporal dependencies in EEG data and effectively reconstructs the corresponding temporal features in the audio.

Furthermore, Pearson correlation value, SSIM, and CW-SSIM metric are computed as shown in Table 9, where Structural Similarity Index (SSIM) and CW-SSIM measure similarity and quality of reconstruction result. On the one hand, the result generated by our approach enjoys a higher Pearson correlation value of 0.0839 than any reconstructed result from other models. On the other hand, our method achieves the highest SSIM of 0.0619 and CW-SSIM of 0.2632. In summary, our approach demonstrates relatively high values in Pearson correlation, SSIM, and CW-SSIM, suggesting better performance in terms of the correlation and structural similarity between the reconstructed speech stimuli and the real speech stimuli.

## 5. Conclusions and Future Work

We proposed an end-to-end training framework incorporating selective augmentation for EEG auditory decoding. To do this, we adopted a self-supervised learning technique to learn the commonality of features before and after augmentation, capturing rich representations of EEG data. We then explored that various augmentation techniques exhibit mutual exclusivity across diverse network architectures and tasks. Therefore, we designed an adaptive augmentation integration selection mechanism to identify the optimal combination of augmentation methods for various models. Furthermore, we proposed an EEG decoding model, NeuroBrain, which merges both local and global information. Additionally, we presented a new auditory EEG dataset, WithMe, intended to facilitate the exploration of the cognitive mechanisms of attention and memory. Our approach yields outstanding performance in reconstruction tasks based on the SparrKULee dataset and in classification tasks on the WithMe dataset. In summary, our work helps to improve the auditory EEG decoding performance across multifarious model architectures on diverse tasks.

Considering the susceptibility of EEG data to noise interference and the inherent differences between EEG and audio data, our methods, although providing impressive improvements, still face challenges in achieving optimal reconstruction performance when decoding EEG data. In future work, we aim to investigate the discriminative relationships between audio and EEG data to enhance the robustness and generalization of EEG decoding models.

## Figures and Tables

**Table 1:** Comparison of Pearson correlation results for different augmentation methods on the SparrKULee dataset. “Original” represents reconstructing from raw EEG signals. “All” denotes integrating all augmentation methods simply. The best results are in bold, and sub-optimal results are underlined.

Augmentation	Linear	VLAAI	FastSpeech2	HappyQuokka	NeuroBrain
Origina	2.933 ± 0.015	4.493 ± 0.000	5.444 ± 0.003	5.441 ± 0.011	**6.903** ± 0.106
Noise	2.933 ± 0.011	3.408 ± 0.213	5.356 ± 0.110	5.311 ± 0.040	**7.135** ± 0.125
Scale	2.941 ± 0.008	4.981 ± 0.186	5.455 ± 0.009	5.351 ± 0.113	**7.096** ± 0.160
Horizontal flipping	2.920 ± 0.011	3.735 ± 0.320	5.383 ± 0.102	5.229 ± 0.136	**6.818** ± 0.268
Vertical flipping	2.915 ± 0.030	2.827 ± 0.371	5.354 ± 0.087	5.175 ± 0.032	**7.056** ± 0.077
Temporal dislocation	2.936 ± 0.007	3.950 ± 0.168	5.377 ± 0.025	5.396 ± 0.107	**7.033** ± 0.027
Time warping	2.920 ± 0.014	3.555 ± 0.404	5.345 ± 0.145	5.341 ± 0.061	**6.955** ± 0.098
Mask	2.946 ± 0.070	4.651 ± 0.310	5.389 ± 0.118	5.315 ± 0.067	**6.970** ± 0.087
All	2.939 ± 0.024	1.471 ± 0.244	0.246 ± 0.153	5.315 ± 0.077	**6.838** ± 0.038

**Table 2:** Classification accuracy on the WithMe dataset: For each evaluated model, the top row indicates “within-subject” classification results, reflecting performance on data from subjects included in the training set. The bottom row denotes the “unseen-subject” classification outcome, showcasing the model’s ability to generalize data from subjects not encountered during training.

Augmentation	EEGNet		NeuroBrain	
	Within-Subject	Unseen-Subject	Within-Subject	Unseen-Subject
Original	79.93% ± 1.14%	76.79% ± 1.23%	85.38% ± 1.65%	78.58% ± 0.97%
Noise	79.76% ± 1.33%	76.33% ± 1.47%	86.59% ± 1.28%	80.29% ± 1.11%
Scale	79.93% ± 1.41%	77.42% ± 1.32%	87.58% ± 0.89%	79.88% ± 1.53%
Horizontal flipping	79.76% ± 1.56%	78.17% ± 1.25%	87.25% ± 1.37%	80.33% ± 1.42%
Vertical flipping	81.05% ± 1.08%	77.13% ± 1.61%	86.97% ± 1.29%	79.46% ± 1.67%
Temporal dislocation	80.58% ± 0.95%	77.52% ± 1.38%	87.66% ± 0.76%	80.25% ± 1.31%
Time warping	79.58% ± 1.62%	77.42% ± 1.34%	87.46% ± 1.21%	78.12% ± 1.85%
Mask	80.66% ± 1.17%	76.33% ± 1.69%	87.68% ± 0.68%	81.58% ± 0.82%
All	80.02% ± 1.44%	76.17% ± 1.77%	86.31% ± 1.48%	80.83% ± 1.26%
Selective	81.85% ± 0.52%	79.75% ± 0.61%	87.68% ± 0.45%	81.58% ± 0.57%

**Table 3:** Statistical test results comparison based on the WithMe dataset for classification task.

Method		Statistical Test Experiment for WithMe Dataset			
Measurement Standard		Value	Value		
Original	Chi-square Test	Pearson Chi-square	862.7	Asymptotic Significance (*p*)	<0.001
		Likelihood Ratio	733.9		
		Linear Association	266.4		
	Correlation Coefficient (Symmetrical Measurement)	Cramer’s V	0.862	Spearman	0.955
		Contingency Coefficient	0.855	Pearson R	0.956
		Gamma	0.994	Kappa	0.840
		Lambda	0.838	Uncertainty Coefficient	0.808
		Cochran’s and Mantel–Haenszel	0.762	Somers’ d	0.925
Noise	Chi-square Test	Pearson Chi-square	812.6	Asymptotic Significance (*p*)	<0.001
		Likelihood Ratio	705.1		
		Linear Association	251.4		
	Correlation Coefficient (Symmetrical Measurement)	Cramer’s V	0.842	Spearman	0.940
		Contingency Coefficient	0.840	Pearson R	0.941
		Gamma	0.991	Kappa	0.818
		Lambda	0.820	Uncertainty Coefficient	0.792
		Cochran’s and Mantel–Haenszel	0.742	Somers’ d	0.906
Scale	Chi-square Test	Pearson Chi-square	845.1	Asymptotic Significance (*p*)	<0.001
		Likelihood Ratio	722.6		
		Linear Association	261.4		
	Correlation Coefficient (Symmetrical Measurement)	Cramer’s V	0.855	Spearman	0.950
		Contingency Coefficient	0.850	Pearson R	0.951
		Gamma	0.993	Kappa	0.832
		Lambda	0.832	Uncertainty Coefficient	0.802
		Cochran’s and Mantel–Haenszel	0.755	Somers’ d	0.918
Horizontal flipping	Chi-square Test	Pearson Chi-square	830.2	Asymptotic Significance (*p*)	<0.001
		Likelihood Ratio	713.8		
		Linear Association	256.5		
	Correlation Coefficient (Symmetrical Measurement)	Cramer’s V	0.848	Spearman	0.945
		Contingency Coefficient	0.845	Pearson R	0.946
		Gamma	0.992	Kappa	0.825
		Lambda	0.826	Uncertainty Coefficient	0.798
		Cochran’s and Mantel–Haenszel	0.748	Somers’ d	0.912
Vertical flipping	Chi-square Test	Pearson Chi-square	912.7	Asymptotic Significance (*p*)	<0.001
		Likelihood Ratio	770.0		
		Linear Association	281.4		
	Correlation Coefficient (Symmetrical Measurement)	Cramer’s V	0.882	Spearman	0.970
		Contingency Coefficient	0.870	Pearson R	0.971
		Gamma	0.997	Kappa	0.865
		Lambda	0.860	Uncertainty Coefficient	0.825
		Cochran’s and Mantel–Haenszel	0.785	Somers’ d	0.945
Temporal dislocation	Chi-square Test	Pearson Chi-square	895.1	Asymptotic Significance (*p*)	<0.001
		Likelihood Ratio	756.4		
		Linear Association	276.3		
	Correlation Coefficient (Symmetrical Measurement)	Cramer’s V	0.875	Spearman	0.965
		Contingency Coefficient	0.865	Pearson R	0.966
		Gamma	0.996	Kappa	0.855
		Lambda	0.852	Uncertainty Coefficient	0.818
		Cochran’s and Mantel–Haenszel	0.775	Somers’ d	0.938
Time warping	Chi-square Test	Pearson Chi-square	795.0	Asymptotic Significance (*p*)	<0.001
		Likelihood Ratio	696.3		
		Linear Association	246.3		
	Correlation Coefficient (Symmetrical Measurement)	Cramer’s V	0.835	Spearman	0.935
		Contingency Coefficient	0.835	Pearson R	0.936
		Gamma	0.990	Kappa	0.812
		Lambda	0.815	Uncertainty Coefficient	0.785
		Cochran’s and Mantel–Haenszel	0.735	Somers’ d	0.900
Mask	Chi-square Test	Pearson Chi-square	930.1	Asymptotic Significance (*p*)	<0.001
		Likelihood Ratio	781.3		
		Linear Association	287.7		
	Correlation Coefficient (Symmetrical Measurement)	Cramer’s V	0.890	Spearman	0.975
		Contingency Coefficient	0.875	Pearson R	0.976
		Gamma	0.998	Kappa	0.880
		Lambda	0.870	Uncertainty Coefficient	0.835
		Cochran’s and Mantel–Haenszel	0.795	Somers’ d	0.955
All	Chi-square Test	Pearson Chi-square	877.6	Asymptotic Significance (*p*)	<0.001
		Likelihood Ratio	745.1		
		Linear Association	271.3		
	Correlation Coefficient (Symmetrical Measurement)	Cramer’s V	0.868	Spearman	0.960
		Contingency Coefficient	0.860	Pearson R	0.961
		Gamma	0.995	Kappa	0.848
		Lambda	0.845	Uncertainty Coefficient	0.812
		Cochran’s and Mantel–Haenszel	0.768	Somers’ d	0.932
Selective	Chi-square Test	Pearson Chi-square	962.5	Asymptotic Significance (*p*)	<0.001
		Likelihood Ratio	812.7		
		Linear Association	295.1		
	Correlation Coefficient (Symmetrical Measurement)	Cramer’s V	0.905	Spearman	0.985
		Contingency Coefficient	0.885	Pearson R	0.986
		Gamma	0.999	Kappa	0.892
		Lambda	0.882	Uncertainty Coefficient	0.845
		Cochran’s and Mantel–Haenszel	0.805	Somers’ d	0.965

**Table 4:** Weight coefficient of seven methods on the SparrKULee dataset. We report the weights of the trainable coefficient of augmentation methods, which are initialized to 0.5 and range from 0 to 1.

Augmentation	Linear	VLAAI	FastSpeech2	HappyQuokka	NeuroBrain
Noise	0.8490	0.5891	0.5502	0.6009	0.7885
Scale	0.8484	0.5892	0.5499	0.6006	0.7884
Horizontal flipping	0.4710	0.4905	0.5023	0.4925	0.4989
Vertical flipping	0.8410	0.5682	0.3442	0.6006	0.7886
Temporal dislocation	0.5596	0.5024	0.4051	0.5093	0.5617
Time warping	0.8483	0.5890	0.5507	0.6007	0.7884
Mask	0.8490	0.5890	0.5525	0.6009	0.7886

**Table 5:** Threshold distribution of the weight coefficient. We report the Pearson correlation of models when the threshold of the coefficient is defined. The negative Pearson correlation loss of one enhanced output will not be calculated if its coefficient is less than the certain threshold set.

Models	Original	0	0.1	0.2	0.3	0.4
linear	2.933	2.948	2.899	2.935	2.938	2.942
VLAAI	4.493	1.519	4.230	4.262	4.381	4.517
FastSpeech2	5.444	1.397	5.304	4.714	0.300	5.272
HappyQuokka	5.441	5.351	5.464	5.365	5.207	5.513
NeuroBrain	6.903	7.010	7.028	7.086	7.012	7.006

**Table 6:** Pearson correlation comparison on different augmentation integration selection strategies. “Original” represents not using augmentation at all. “All” denotes integrating all augmentation methods simply. “Manual” means selecting good ones with a high weight coefficient.

Integration Strategy	Linear	VLAAI	FastSpeech2	HappyQuokka	NeuroBrain
Original	2.933	4.493	5.444	5.441	6.903
All	2.939	1.471	0.246	5.351	6.838
Manual	2.926	4.358	5.006	5.197	7.014
Selective	2.960	4.981	5.455	5.513	7.135

**Table 7:** The performance on SparrKULee with different training sample sizes.

Sample Size	Average Pearson	Standard Deviation
30%	4.80	0.33
70%	8.02	3.68
100%	12.35	0.76

**Table 8:** Performance comparison in the ablation studies. “Improvement” represents the performance improvement of the model relative to the baseline.

Models	Pearson Correlation	Improvement (%)
baseline (HappyQuokka)	5.513	-
NeuroBrain	7.135	29.42
*w*/*o* augmentation	6.903	25.21
*w*/*o* contrast learning	6.988	26.75
*w*/*o* cross attention	6.828	23.85
*w*/*o* selection	6.838	24.03

**Table 9:** Similarity between reconstruction results and ground-truth speech stimuli. We report Pearson correlation, SSIM, and CW-SSIM of reconstructed speech and real speech.

Models	Pearson Correlation	SSIM	CW-SSIM
Linear	0.48	1.97	19.02
VLAAI	2.38	4.28	23.95
FastSpeech2	5.91	3.44	23.12
HappyQuokka	0.22	1.79	12.04
NeuroBrain	8.39	6.19	26.32

## Data Availability

The dataset can be accessed at https://exporl.github.io/auditory-eeg-challenge-2024/dataset/ (accessed on 2 January 2026).
